# Tobramycin at subinhibitory concentration inhibits the RhlI/R quorum sensing system in a *Pseudomonas aeruginosa *environmental isolate

**DOI:** 10.1186/1471-2334-10-148

**Published:** 2010-06-02

**Authors:** Fedora Babić, Vittorio Venturi, Gordana Maravić-Vlahoviček

**Affiliations:** 1 Department of Biochemistry and Molecular Biology, Faculty of Pharmacy and Biochemistry, University of Zagreb, Ante Kovačića 1, 10000 Zagreb, Croatia; 2 Bacteriology Group, International Centre for Genetic Engineering and Biotechnology, Area Science Park, Padriciano 99, 34149 Trieste, Italy

## Abstract

**Background:**

Antibiotics are not only small molecules with therapeutic activity in killing or inhibiting microbial growth, but can also act as signaling molecules affecting gene expression in bacterial communities. A few studies have demonstrated the effect of tobramycin as a signal molecule on gene expression at the transcriptional level and its effect on bacterial physiology and virulence. These have shown that subinhibitory concentrations (SICs) of tobramycin induce biofilm formation and enhance the capabilities of *P. aeruginosa *to colonize specific environments.

**Methods:**

Environmental *P. aeruginosa *strain PUPa3 was grown in the presence of different concentrations of tobramycin and it was determined at which highest concentration SIC, growth, total protein levels and translation efficiency were not affected. At SIC it was then established if phenotypes related to cell-cell signaling known as quorum sensing were altered.

**Results:**

In this study it was determined whether tobramycin sensing/response at SICs was affecting the two independent AHL QS systems in an environmental *P. aeruginosa *strain. It is reasonable to assume that *P. aeruginosa *encounters tobramycin in nature since it is produced by niche mate *Streptomyces tenebrarius*. It was established that SICs of tobramycin inhibited the RhlI/R system by reducing levels of C4-HSL production. This effect was not due to a decrease of *rhlI *transcription and required tobramycin-ribosome interaction.

**Conclusions:**

Tobramycin signaling in *P. aeruginosa *occurs and different strains can have a different response. Understanding the tobramycin response by an environmental *P. aeruginosa *will highlight possible inter-species signalling taking place in nature and can possible also have important implications in the mode of utilization for human use of this very important antibiotic.

## Background

Aminoglycoside antibiotics are clinically very important therapeutics widely used for treatment of chronic bacterial infections caused by Gram-negative bacteria. They bind to a specific domain of the prokaryotic 30 S ribosomal subunit and interfere with protein synthesis causing either inhibition of cell growth or cell death [1,2]. For example, tobramycin, an aminoglycoside produced by *Streptomyces tenebrarius *is commonly used for its effectiveness against opportunistic cystic fibrosis (CF) infections of *Pseudomonas aeruginosa *[3]. Tobramycin affects protein synthesis by binding to the A site of the 30 S ribosomal subunit [4], interacting with the A1408 residue (*E. coli *numbering) of the decoding site 16 S rRNA [5].

It has recently been reported, that antibiotics are not only small molecules with therapeutic activity in killing or inhibiting microbial growth, but can also act as signaling molecules affecting gene expression in bacterial communities [6-9]. Bacterial signaling molecules are defined as being able to (i) elicit a response unrelated to the metabolism or detoxification of the signaling molecule, (ii) accumulate extracellularly, (iii) generate a coordinated response once a critical concentration is reached. Antibiotics meet these criteria and now a few studies are beginning to lay the foundation that antibiotics can modulate global transcription also by affecting/interfering with known global cascade responses [10-12]. This signaling activity occurs at much lower concentrations than those required for antibiosis being as low as 1% or as high as 50% of those required for inhibition (which is referred to as minimal inhibitory concentration or MIC) depending on the compound. The concentration at which antibiotic signaling is studied are in subinhibitory antibiotic concentrations, or SICs. SIC of antibiotics have been shown to affect several phenotypes in diverse bacteria through induced transcription modulation including virulence functions in group A streptococci [13], virulence and motility in *Salmonella typhimurium *[9] and biofilm formation in *Pseudomonas aeruginosa *[14].

A few studies have demonstrated the effect of tobramycin as a signal molecule on gene expression at the transcriptional level [10,11] and its effect on bacterial physiology and virulence [14]. These studies have shown that low SICs of tobramycin induce biofilm formation [14] and enhance the capabilities of *P. aeruginosa *to colonize specific environments [11]. The response to SICs has therefore, important implications on the antibiotic therapy of *P. aeruginosa *infections, but the molecular mechanisms governing this response are currently unknown.

In *P. aeruginosa*, the regulation of many virulence associated factors, as well as biofilm formation, are under regulation of two hierarchically arranged *N*-acyl homoserine lactone (AHL) quorum-sensing (QS) systems, namely the LasI/R and RhlI/R systems [3]. Each system contains one gene encoding AHL sensor/transcriptional regulator, i.e. *lasR *and *rhlR*, and a gene encoding an autoinducer synthase, i.e. *lasI *and *rhlI*, required for the synthesis of the autoinducer molecules *N*-(3-oxo-dodecanoyl)-L-homoserine lactone (3-oxo-C12-HSL) and *N*-(butanoyl)-L-homoserine lactone (C4-HSL), respectively (reviewed by [3,15,16]). At high cell density in a situation of quorum AHL concentrations, 3-oxo-C12-HSL and C4-HSL interact directly with the cognate LasR and RhlR sensor/regulator affecting transcription of target genes.

Since QS is a global regulatory mechanism coordinated by bacterial communities, scientists have begun to investigate the possible link with antibiotic signalling. For example the response of *P. aeruginosa *to the macrolide antibiotic azithromycin studied by microarray, proteomics and phenotype analysis revealed that there is a large common subset of genes regulated by QS and azithromycin [14,17,18]. These studies demonstrated that azithromycin has considerable QS-antagonistic effects thus when applied it improves *P. aeruginosa *infections of CF patients. In fact, SIC of azithromycin were shown by microarray analysis to repress a large number of genes which are QS regulated and similar observations were made with other antibiotics [12]. In this study we were interested to determine whether tobramycin sensing/response at SICs was affecting the two AHL QS systems in *P. aeruginosa*. In this study we used an environmental isolate of *P. aeruginosa *PUPa3 in which the two AHL QS systems were not hierarchically organized. The reason for performing these studies using an environmental isolate of *P. aeruginosa *was because the producer of tobramycin, *Streptomyces tenebrarius*, lives in similar niches of *P. aeruginosa *hence it was of interest to determine if inter-species signalling could take place between them.

## Methods

### Bacterial strains, plasmids and growth conditions

Strains, plasmids and primers used in this study are listed in Table 1. *P. aeruginosa *PUPa3 has been previously isolated from the rice rhizosphere in India [19]. For DNA transformations *E. coli *DH5α was used [20] while for triparental matings we used *E. coli *DH5α (pRK2013) as helper [21]. Cultures were grown at 37°C aerobically in Luria-Bertani (LB) broth or 1.5% agar (Bacto-agar, BD Difco) plates containing appropriate antibiotics. *P. putida *was grown at 30°C. Antibiotics were added when required at the following final concentrations: ampicillin 100 μg/mL, tetracycline 15 μg/mL (*E. coli*) or 100 μg/mL (*Pseudomonas*), gentamicin 100 μg/mL (*Pseudomonas*); kanamycin 100 μg/mL, chloramphenicol 25 μg/mL (*E. coli*) or 250 μg/mL (*Pseudomonas*).

**Table 1:** Strains, plasmids and primers used.

Strains, Plasmids and Primers	Characteristics	Reference or source
**Strains**		
***Pseudomonas aeruginosa***		
**PUPa3**	Wild type, rice rhizosphere isolate	[42]
**LASI**	*lasI*::Km of *P. aeruginosa *PUPa3; Km^r^	[43]
**RHLI**	*rhlI*::Gm of *P. aeruginosa *PUPa3; Gm^r^	[43]
**DMI**	*lasI*::Km *rhlI*::Gm of *P. aeruginosa *PUPa3; Km^r ^Gm^r^	[43]
***Pseudomonas putida***		
**SM17**	*rsaL*::Tc of *P. aeruginosa *PUPa3; Tc^r^	[43]
***E. coli***		
**M15(pRep4)**	Derivative of *E. coli *K-12, containing pREP4 plasmid ensuring the production of high levels of *lac *repressor protein; Km^r^	Qiagen
**DH5α**	F'/*endA1 hsdR17 supE44 thi-1 recA1 gyrA relA1 (lacZYA-argF)U169 deoR *[80d*lac(lacZ)*M15*recA1*]	[44]
**Plasmids**		
**pMP220**	Promoter probe vector, IncP1; Tc^r^	[45]
**pMOSBlue**	Cloning vector; Amp^r^	Amersham-Pharmacia
**pGEM-T Easy**	Cloning vector; Amp^r^	Promega
**pBBRmcs3**	Broad-host-range vector; Tc^r^	[46]
**pQE30**	Expression vector, Amp^r^	Qiagen
**pRTLF-1**	*rpoS-lacZ *translational fusion in pMP77; Cm^r^	[47]
**pSB406**	contains a fusion of *rhlRI'::luxCDABE *in pUC18; Amp^r^	[48]
**pSB536**	*ahyR^+ ^ahyI::luxCDABE*; ColE1 origin; Amp^r^	[49]
**pRK2013**	Tra^+ ^Mob^+ ^ColE1 replicon; Km^r^	[50]
**pRPO220**	pMP220 containing promoter *rpoS*; Tc^r^	[51]
**pRSAL220**	pMP220 containing promoter *rsaL*; Tc^r^	[47]
**pRHLI**	pMP220 containing promoter *rhlI*; Tc^r^	This study
**pLASI**	pMP220 containing promoter *lasI*; Tc^r^	This study
**pQERHLR**	pQE30 containing *rhlR*; Amp^r^	This study
**pRMTA**	pBBR3 containing *rmtA*; Tc^r^	This study
**pRMTC**	pBBR3 containing *rmtC*; Tc^r^	This study
**Primers**		
**RhlR-fw**	GGGGTACCAGGAATGACGGAGGCTTTTTG	This study
**RhlR-rv**	GAAGCTTGATGAGGCCCAGCGCCGCGG	This study
**rhlI-fw**	CCGAATTCCACCACAAGAACATCC	This study
**rhlI-rv**	ATGGTACCAGCGATTCAGAGAGCAA	This study
**lasI-fw**	GGAATTCGGGCTGTGTTCTCTCGTGTG	This study
**las-rv**	CTCTAGAGAACTCTTCGCGCCGACCAA	This study
**RmtA-fw**	GGGTACCAATAATTTTGTTTAACTTTA	[52]
**RmtA-rv**	CCCTCGAGTCACTTATTCC	This study
**RmtC-fw**	ATCTGCAGAATAATTTTGTTTAACTTTA	This study
**RmtC-rv**	ATACTAGTTTACAATCTCGATACGAT	This study

Ap^r^, Km^r^, Tc^r^, Gm^r^, and Cm^r^, resistant to ampicillin, kanamycin, tetracycline, gentamicin, and chloramphenicol, respectively

### DNA manipulations and plasmid constructs

Recombinant DNA techniques, including digestion with restriction enzymes, agarose gel electrophoresis, purification of DNA fragments, ligation with T4 ligase and transformation of *E. coli *were performed as described [22]. Plasmids pLASI and pRHLI were constructed as follows. The *lasI *gene and *rhlI *gene promoters were amplified using *P. aeruginosa *PUPa3 chromosomal DNA as template and using primers lasI-fw/lasI-rv and rhlI-fw/rhlI-rv respectively, listed in Table 1. PCR fragments were ligated in pMOSBlue and digested with EcoRI and XbaI or EcoRI and KpnI respectively and then ligated in the *lacZ *promoterless cloning vector pMP220, yielding pLASI and pRHLI respectively. The *rhlR *gene was amplified from PUPa3 chromosomal DNA and ligated into pGEM cloning vector, then digested with KpnI and HindIII and ligated into the corresponding sites of pQE30 generating pQERHLR. Methyltransferase genes *rmtA *and *rmtC *were kindly provided from Dr. Jason P. Rife (University of North Carolina, USA) as expression vectors pET-15b(+) with coding genes for *rmtA *and *rmtC *respectively. The *npmA *gene synthesis was ordered from the Epoch Biolabs, INC. following the sequence published in [23]. The gene was subsequently cloned in pBBRmcs3 with restriction sites XbaI and SacI generating pNPMA. *rmtA *and *rmtC *were cloned in pET-25b(+) vectors with restriction sites NdeI and XhoI after PCR amplification using primers RmtA-fw/RmtA-rv and RmtC-fw/RmtC-rv respectively. PCR fragments were then ligated in pMOSBlue, digested with KpnI and XhoI or PstI and SpeI respectively and ligated in pBBRmcs3 generating pRMTA and pRMTC. All plasmid constructs were verified by DNA sequencing (Macrogen, Korea).

### Determination of Minimal Inhibitory Concentration (MIC)

The minimal inhibitory concentration (MIC) of tobramycin (Sigma-Aldrich) for *P. aeruginosa *PUPa3 was determined in LB broth after 24 hours incubation at 37°C using the MIC dilution method as previously described [24]. Briefly, overnight cultures of PUPa3 were diluted 100 fold in fresh LB medium and grown until the OD_600 _of 1. LB medium containing various concentrations of tobramycin (0.05 - 320 μg/mL) was inoculated with 10^6 ^cells and grown in microtiter plates. MIC was determined as a minimal concentration of tobramycin that prevents the visible growth of bacteria.

### Determination of growth rate and of total cell protein and β-galactosidase assay

Growth curves were performed in duplicates in LB or LB medium supplemented with various concentrations of tobramycin (1/16, 1/8, 1/4, 1/2 of MIC). Overnight culture of *P. aeruginosa *PUPa3 was diluted 100 fold in fresh LB medium and cultures were grown at 37°C measured the OD_600 _every 20 minutes. β-galactosidase activities were determined by the Miller method [22] and levels of proteins were determined by the Bradford assay [25].

### Determination of swarming ability, proteolytic activity and biofilm formation

Swarming assay was performed on 0.5% LB agar plates as previously described [26] supplemented with SIC of tobramycin (25% of MIC). 1.5 μl of overnight culture of PUPa3 was inoculated on swarming plates and incubated at 37°C for 24 hours. Synthetic C4-HSL (purchased from Fluka-Sigma-Aldrich) was added in agar plates in final concentration of 2 μM. Proteolytic activity was determined in the appropriate indicator plates as previously reported [27] in presence or absence of 25% of MIC of tobramycin and grown at 37°C after 24 hours.

Biofilm formation was quantified by measuring A_570 _of crystal-violet staining of adherent cells. 2 ml of overnight culture from *P. aeruginosa *PUPa3 grown in AB minimal medium was centrifuged and pellet was washed in fresh media. New inoculum of OD_600 _of 0,01 was grown at 37°C for 24 hours in wells of 96-well polystyrene microtiter plates (Nunc) using 100 μl of culture per well (16 duplicates per condition per experiment). Planktonic cells were removed and the wells with adherent biofilms were stained using 0,1% crystal-violet for 30 min at room temperature, washed three times with water and dried. To solubilize adsorbed crystal violet, 150 μl of DMSO was added in each well and incubated for 20 minutes. The absorbance was read at 570 nm. Unpaired t-test and ANOVA test (Prisma 5.5 software, GraphPad Software, San Diego, California) was used to establish significance of differences between means (P value of < 0,005). MIC was determined for the planktonic cells in the culture used as the inoculum. MIC of tobramycin was 8 μg/ml and 25% of MIC was used as subinhibitory concentration.

### Extraction of AHL signal molecules

For the extraction of the AHL molecules, *P. aeruginos*a PUPa3 was grown until OD_600 _reached 2 in 50 ml of LB medium or LB medium supplemented with SIC of tobramycin, and supernatant of the culture was extracted with an equal volume of ethyl acetate acidified with 0.1% acetic acid. The extract was then dried and resuspended in a small volume of ethyl acetate as previously described [26].

### Determination of C4-HSL, of 3-oxo-C12-HSL and of pyocyanin levels

C4-HSL levels were determined as follows. Bioluminescence assay was performed using biosensors specific for C4-HSL, i.e. *E. coli *pSB406 [28] and *E. coli *pSB536 [29]. Overnight liquid cultures of the two reporter strains were diluted 1:100 in LB and grown till OD_600 _of 1 then added to the wells of black clear-bottom 96-well plates (OptiPlate microtiter trays, Perkin-Elmer, USA) and supplemented with various concentrations of synthetic C4-HSL to generate standard curve or with various amounts of *P. aeruginosa *AHL extracts. Cultures were then grown at 37°C and OD_600 _and luminescence from each well were recorded in a Wallac EnVision 2104 multilabel reader (Perkin-Elmer). Relative light intensity of extracts of *P. aeruginosa *grown in the presence of tobramycin was defined as the percentage of emitted light (British light units) under these conditions compared with the mean value of *P. aeruginosa *extracts grown without antibiotic.

3-oxo-C12-HSL was determined as previously described [30]. Briefly, β-galacotsidase activities were measured using *P. putida *SM17 (pRSAL220) *lacZ *based reporter strain upon addition of PUPa3 AHL extracts. Overnight cultures of *P. putida *were diluted up in 5 ml volume to an OD_600 _of 0.3 and *P. aeruginosa *AHL extracts were added. β-galactosidase activity was measured during various stages of growth. All assays were performed in triplicate.

Pyocyanin levels were determined as previously described [31]. Briefly, *P. aeruginosa *PUPa3 was grown with aeration at 37°C in LB broth. Each culture was inoculated from a fresh overnight culture adjusted an optical density at 600 nm of 0.02 before incubation. Quantification of pyocyanin production was done by extracting a 5 ml of culture with 3 ml of chloroform followed by mixing with 1 ml of 0.2 M HCl. The absorbance of upper red phase was measured at 520 nm and amount of pyocyanin was determined as previously described [31].

## Results

### MIC and SIC of tobramycin for *P. aeruginosa *PUPa3

The MIC of tobramycin for strain PUPa3 was determined to be 0.2 μg/mL in LB broth and 2 μg/mL on LB agar plates. We then established that 25% of the MIC of tobramycin (i.e. 0.05 μg/mL) was subinhibitory (SIC), since (i) it did not affect the growth rate of *P. aeruginosa *PUPa3 in LB media (data not shown), (ii) did not alter the protein synthesis as determined by measurement of total cellular protein (data not shown) and (iii) it did not affect translation efficiency because did not alter β-galactosidase activity of PUPa3 harboring the *rpoS-lacZ *translational fusion at various growth stages (data not shown).

### SIC of tobramycin affects swarming motility, biofilm formation and pyocyanin production in *P. aeruginosa *PUPa3

**Figure 1:**
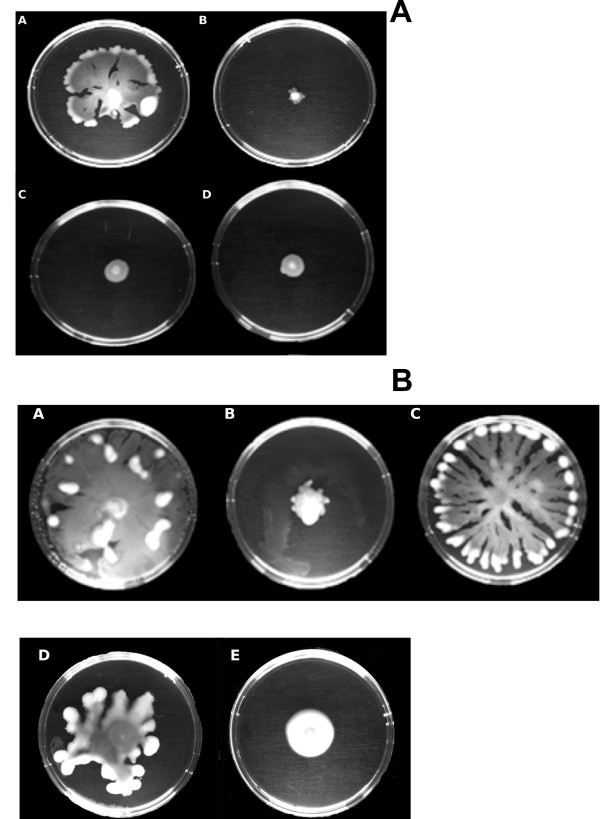
**Effect of subinhibitory concentration of tobramycin on swarming motility in *P. aeruginosa *PUPa3**. **a) **Swarming of *P. aeruginosa *PUPa3 on 0.5% LB agar plates at 37°C after 24 hrs. WT swarms (A), but in presence of SIC of tobramycin (0.05 μg/mL) cannot swarm (B). Swarming was not present in the *rhlI *mutant (C) nor in double mutant *lasI/rhlI *(D). **b) **Swarming of *P. aeruginosa *PUPa3 on 0.5% LB agar plates at 37°C after 40 hrs. Normal swarming occurs in WT (A), no swarming is present in WT on plate supplemented with SIC of tobramycin (B) and swarming was restored upon the addition of 2 μM exogenous synthetic C4-HSL (C). Swarming in the *rhlI *mutant was also restored upon the addition of 2 μM exogenous synthetic C4-HSL (D), but not upon the addition of 2 μM exogenous synthetic 3-oxo-C12-HSL (E).

**Figure 2:**
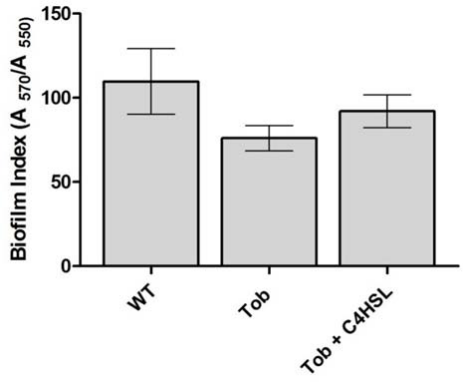
**Effect of subinhibitory concentration of tobramycin on biofilm growth**. Biofilm formation was reduced when PUPa3 was grown under subinhibitory concentration of tobramycin (Tob). Biofilm formation was rescued by adding 2 μM exogenous C4-HSL (Tob + C4HSL).

**Figure 3:**
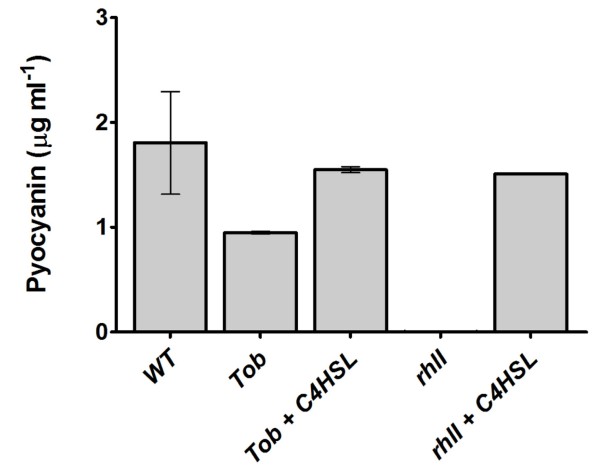
**Pyocyanin production in PUPa3 in early stationary growth phase**. All strains were grown in LB media. Presence of SIC of tobramycin in wild type significantly decreases levels of pyocyanin production (P < 0.05). Values shown in graph are means from three independent biological experiments, normalized to culture density (OD_600_). WT, is strain PUPa3; Tob, is strain PUPa3 grown in the presence of SIC of tobramycin (0.05 μg/mL); Tob C4HSL, is strain PUPa3 grown in the presence of SIC of tobramycin (0.05 μg/mL) and 2 μM of C4-HSL; *rhlI*, is the *rhlI *knock-out mutant of strain PUPa3; *rhlI *C4HSL, is the *rhlI *knock-out mutant of strain PUPa3 grown in the presence of exogenously added 2 μM of C4-HSL.

It was of interest to determine the impact of SIC of tobramycin on the AHL QS response in *P. aeruginosa *PUPa3 using swarming assays. It was previously established that in *P. aeruginosa *PUPa3 swarming is regulated by the RhlI/R AHL QS system [26]. We observed as expected that the *rhlI *and double mutant (*lasI/rhlI*) were impaired in swarming whereas strain PUPa3 swarmed well. We then tested swarming abilities at SIC and established that strain PUPa3 did not swarm under these conditions indicating that 0.05 μg/mL of tobramycin impaired swarming (Figure 1a). Importantly, swarming can then be rescued by adding exogenous synthetic C4-HSL, indicating that possibly the presence of SIC of tobramycin affected the production levels C4-HSL (Figure 1b). Biofilm formation was also reduced when *P. aeruginosa *strain PUPa3 grows under SIC of tobramycin; this reduction could then be rescued by the exogenous addition of C4-HSL (Figure 2). Another phenotype previously reported to be regulated by the RhlI/R system is the production of the toxic pyocyanin pigment [32]. We also assayed for pyocyanin production (Figure 3) and determined that (i) the *rhlI *mutant of *P. aeruginosa *PUPa3 no longer produced pyocyanin and (ii) SIC concentrations of tobramycin reduced pyocyanin production which could be also rescued by addition of exogenous C4-HSL (Figure 3). It was therefore concluded that pyocyanin in strain PUPa3 is regulated by the RhlI/R system and that tobramycin affected the production levels.

**Figure 4:**
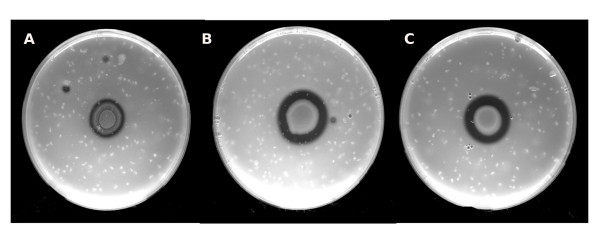
**Proteolytic activities determined in the appropriate indicator LB plates**. The *P. aeruginosa lasI *mutant (A) showed four-fold reduced proteolytic activity compared to WT (B). WT shows no difference in proteolytic activity in the presence of 0.05 μg/mL SIC of tobramycin (C).

In order to establish whether SIC of tobramycin affected the LasI/R AHL QS system of strain PUPa3, we assayed for protease production since as previously determined, protease activity is regulated by LasI/R [26]. It was established that when *P. aeruginosa *was grown in the presence of SIC of tobramycin proteolytic activity was unaltered when compared to *P. aeruginosa *grown in the absence of the antibiotic (Figure 4). It was therefore concluded that the LasI/R system was not affected by SIC of tobramycin.

### *P. aeruginosa *PUPa3 grown in SIC of tobramycin lowers C4-HSL levels

**Figure 5:**
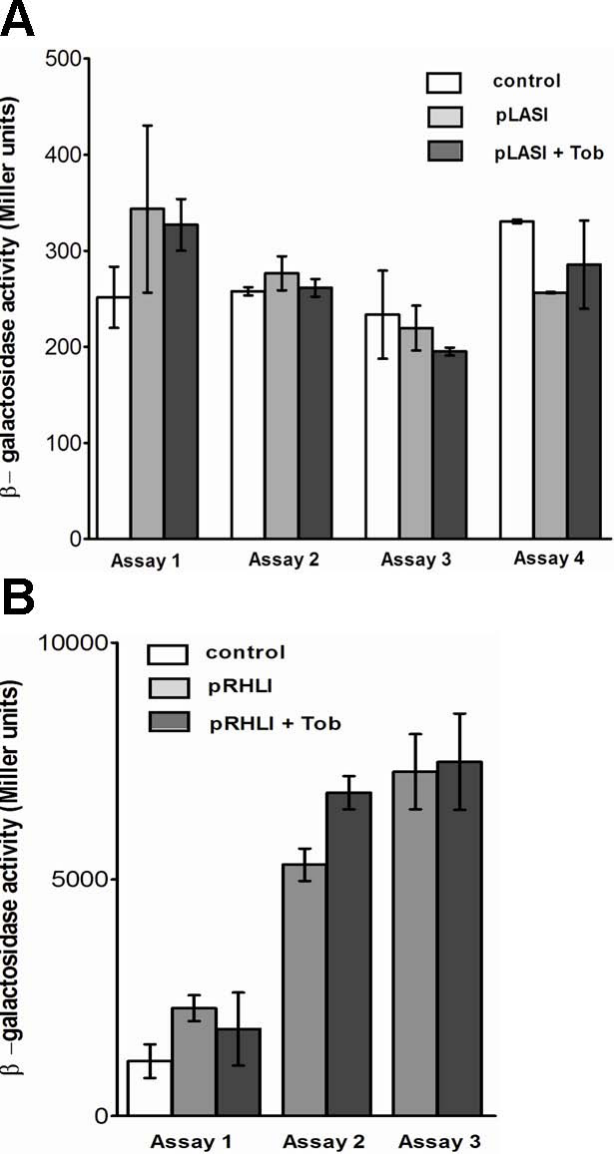
***lasI *****and *****rhlI *****gene promoter activities and quantitative determination of 3-oxo-C12-HSL. a)***lasI *gene promoter activities measured via β-galactosidase assay in PUPa3 in different stages of growth. The white colorless bars represents the control culture *P. aeruginosa *PUPa3 with the vector pMP220; light grey represents *P. aeruginosa *PUPa3 harboring pLASI; dark grey, represents *P. aeruginosa *PUPa3 harboring pLASI with addition of SIC of tobramycin (0.05 μg/mL). **b) ***rhlI *gene promoter activities measured via β-galactosidase assay in PUPa3 in different stages of growth. The white colorless bars represents the control culture *P. aeruginosa *PUPa3 with the vector pMP220; light grey represents *P. aeruginosa *PUPa3 harboring pRHLI; dark grey, represents *P. aeruginosa *PUPa3 harboring pRHLI with addition of SIC of tobramycin (0.05 μg/mL). The β-galactosidase activities were performed at different stages of growth: assay 1 was performed during log phase, assay 2 during early stationary phase, assay 3 and assay 4 during stationary phase. Each assay was performed in biological triplicates. Error bars represent the standard deviations calculated from at least three separate experiments.

**Figure 6:**
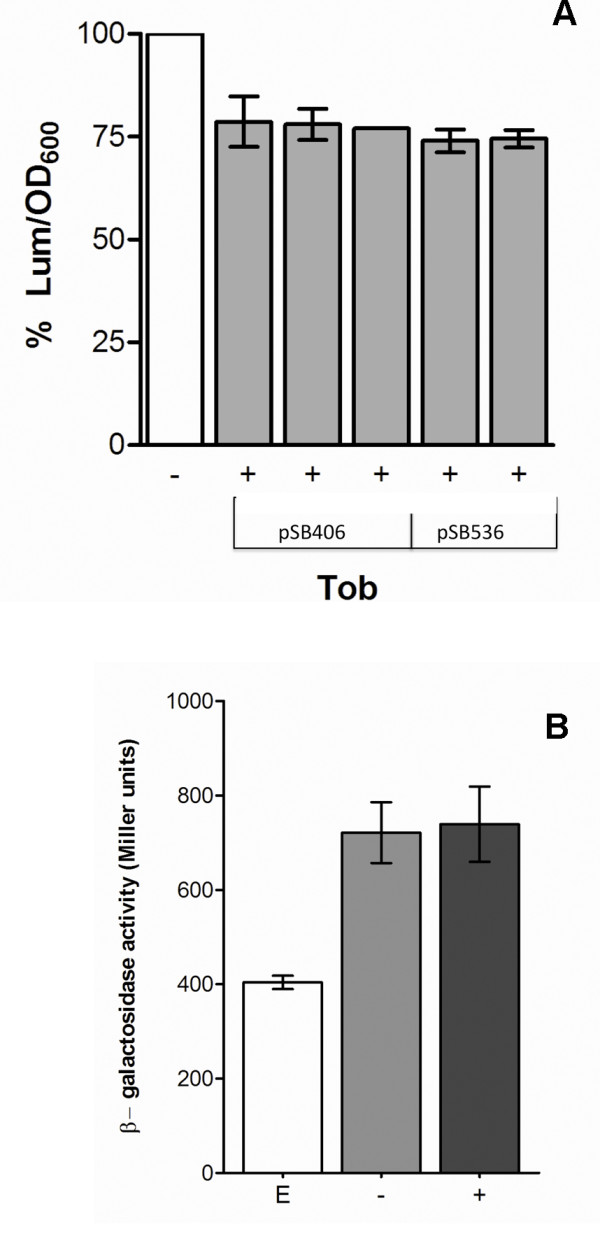
**C4-HSL and 3-oxo-C12-HSL quantification**. **a) **C4-HSL quantification using two *E. coli luxCDABE *specific C4-HSL biosensors harbored in pSB406 and pSB536. Bioluminescence induced by AHL extracts of PUPa3 corresponding to 7 × 10^9 ^cells grown in presence of SIC of tobramycin (0.05 μg/mL) at 37°C was defined as the percentage relative to the same strain without antibiotic (white bar). Each assay was an independent experiment measured in triplicates. +/- represents extract from PUPa3 grown in presence or absence of SIC of tobramycin added to biosensor culture. **b) **3-oxo-C12-HSL quantification: the columns represent the 3-oxo-C12-HSL quantification determined by using the biosensor *P. putida *SM17 (pRSAL220). E represents 5 ml of biosensor culture supplemented with 5 μl ethyl-acetate, +/- addition of 5 μl of AHL extract of PUPa3 (equivalent to 9.2 × 10^9 ^cells) grown in presence or absence of SIC of tobramycin (0.05 μg/mL).

In order to establish whether SIC of tobramycin affected AHL levels we first assayed *rhlI *and *lasI *transcription via a *rhlI-lacZ *and *lasI-lacZ *transcriptional gene promoter fusions. β-galactosidase activity in strain PUPa3 harboring either the plasmid pRHLI or pLASI showed no significant differences at various stages of growth in presence of SIC of tobramycin when compared to PUPa3 grown without tobramycin (Figure 5). It was therefore concluded that SIC of tobramycin did not affect the transcription rates of the *rhlI *and *lasI *AHLs synthases. Levels of 3-oxo-C12-HSL from PUPa3 extracts were then measured using the *P. putida *SM17 (pRSAL220) specific 3-oxo-C12-HSL sensor and no significant difference has been detected between cultures of PUPa3 of different growth stages grown in presence or absence of SIC of tobramycin (Figure 6b). Two *E. coli luxCDABE *based biosensors specific to C4-HSL were used in order to establish C4-HSL levels. Bioluminescence assays demonstrated that a significant reduction (20 - 25%) of C4-HSL was observed when *P. aeruginosa *PUPa3 grown in presence of SIC of tobramycin (Figure 6a). It was concluded that SIC of tobramycin did not affect 3-oxo-C12-HSL levels but did lower C4-HSL levels.

### SIC of tobramycin response requires interaction with ribosome

**Figure 7:**
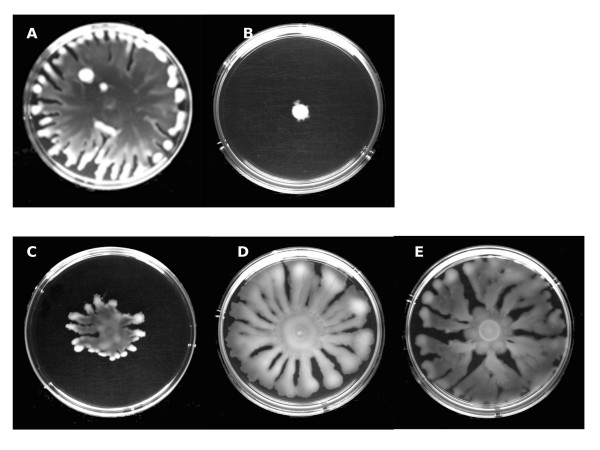
**Swarming of *P. aeruginosa *PUPa3 on 0.5% LB agar plates at 37°C after 24 hrs**. In the presence of SIC tobramycin (0.05 μg/mL) no swarming is present in WT (B), but the swarming was restored with the expression of RmtA (C), RmtC (D) or NpmA (E) methyltransferases as in LB plates inoculated with WT (A).

In order to determine the importance of tobramycin-ribosome interaction in the signaling role in lowering C4-HSL levels, the *npmA, rmtA *and *rmtC *methyltransferase genes were cloned and expressed in *P. aeruginosa *PUPa3. These methyltransferases are known to specifically methylate nucleotides A1408 [23] and G1405 [33,34] respectively, within the decoding A site in small ribosomal subunit which then block tobramycin-ribosome interaction rendering the bacterium resistant to tobramycin. As observed above, *P. aeruginosa *PUPa3 could not swarm in the presence of SIC of tobramycin but when either the *npmA *or *rmtA *or *rmtC *genes were expressed using plasmids pNPMA, pRMTA and pRMTC respectively, swarming was restored (Figure 7). It was concluded that the observed effect of SIC of tobramycin in affecting C4-HSL dependent swarming involves tobramycin-ribosome interaction.

## Discussion

It is important for us bacteriologists to remember that most bacterial species in any given environment encounter a huge mixed population of prokaryotes meaning that bacteria do not live in isolation or in monoculture but in mixed communities. It is also most probable that in these mixed and diverse consortia there is considerable competition as well as constant communication. It is assumed that antibiotics with inhibitory activity are important as weapons in inter-microbial competition. In recent years however scientists are beginning to investigate that antibiotics have functional interactions with cellular processes which are different from the primary antibiotic inhibitory effect (reviewed by [7,9]). Several studies have now shown that subinhibitory concentrations (SICs; these do not affect growth and protein synthesis) of several antibiotics can transcriptionally modulate a large number of genes [10-12].

Here we began to study the effect of SICs of tobramycin on an environmental isolate of *P. aeruginosa*. Tobramycin is produced by *Streptomyces tenebrarius *which is a soil organism and therefore probably shares similar niches to *P. aeruginosa *since it is also found in the soil. *P. aeruginosa *in nature therefore encounters tobramycin and could have evolved the ability to respond to this molecule possibly allowing adaptation to co-exist or compete with *S. tenebrarius*. Importantly, *P. aeruginosa *possesses an inducible resistance to tobramycin [35] thus suggestive of a response and adaptive behavior towards the compound. Since *P. aeruginosa *possesses a major transcription response to AHL signals influencing the transcription of over 6% of the genome [16], it was of interest to determine if AHL QS was affected by SICs of tobramycin. The *P. aeruginosa *PUPa3 environmental isolate possesses the two AHL QS systems which act independently and are not hierarchically organized [26]. To our knowledge all other studies in *P. aeruginosa *involving antibiotics as signal molecules were performed using the clinical isolates. In fact it was previously reported that SIC of tobramycin in strain *P. aeruginosa *PAO1 (clinical isolate of 1950) also induces a reduction in C12-3-oxo-HSL and C4-HSL production [36]. It must be noted however that the two AHL systems in strain PAO1 are organized in a hierarchy; the Las system regulating in a cascade the *rhl *system [37]. SICs of tobramycin in strain PUPa3 reported here was found to lower only the C4-HSL levels by over 20% which was shown to be enough to inhibit swarming and lower pyocyanin levels which are both regulated by the RhlI/R system. Importantly both of these effects were recovered upon addition of exogenous C4-HSL indicating that they were entirely due to interference with the RhlI/R system. Importantly in strain PUPa3 the levels of C12-3-oxo-HSL were not affected by SICs of tobramycin possibly indicating that the independent organization of the two systems allows them to respond differently to environmental conditions. Another study involving *P. aeruginosa *PAO1, has also reported effects by tobramycin signaling. Linares et al. [11], have reported that SIC of tobramycin induces swarming as well as biofilm formation in *P. aeruginosa *PAO1. This is contrast of what we report here and what was reported by Luke et al. [36] since we observed a decrease in swarming and biofilm formation when *P. aeruginosa *PUPa3 is exposed to SICs of tobramycin. It is most likely that tobramycin signaling can have different effects in different strains when grown in different growth conditions. In fact another recent study reported that SICs of tobramycin in *P. aeruginosa *PAO1 did not inhibit AHL QS [12] as we observe for strain PUPa3. Three antibiotics however were reported to inhibit AHL QS at SIC in *P. aeruginosa *PAO1 [12]; interestingly these antibiotics, namely floroquinolone, cephalosporin and azithromycin, belong to different classes. The inhibition of the RhlI/R system in strain PUPa3 by SIC of tobramycin observed here is similar to what was reported for azithromycin signaling in strain PAO1, however in PAO1 both Las and Rhl systems are inhibited [18,38,39]. Interestingly this inhibition is also not due to a decrease in the transcription of the AHL QS systems but apparently to an effect on loci upstream the *lasI/R *and *rhlI/R *systems [38]. It therefore appears that there are some common features between the signaling response of strain PAO1 to azithromycin and tobramycin signaling in strain PUPa3.

How does SIC of tobramycin affect the RhlI/R system? The fact that the LasI/R system was not affected showed that the SIC of tobramycin effect is specific for the RhlI/R system. Since a few studies have reported that SIC of several antibiotics affected gene transcription, we analyzed whether *rhlI *transcription levels changed at SICs of tobramycin. The *rhlI *transcription levels did not change upon exposure to SIC of tobramycin at various growth stages indicating that the lower C4-HSL levels were not due to a decrease of *rhlI *transcription. It cannot be excluded that this could be due to an effect on *rhlI *translation; recently it has been reported that a novel protein designated GidA, post-transcriptionally controls RhlI/R levels [40]. In addition, tobramycin at subinhibitory concentrations could result in lower pools of the precursors necessary for C4-HSL biosynthesis or possibly the transport of the AHL could also be affected. The C4-HSL however has been shown that when added to cell suspensions of *P. aeruginosa*, the cellular concentration reached a steady state in less than 30 seconds being equal to the external concentration, hence it can be regarded as a freely diffusible compound [41]. Future studies need to determine the cellular targets which are affected by subinhibitory concentrations of tobramycin which result in lower C4-HSL levels in *P. aeruginosa *PUPa3. Previous studies in *P. aeruginosa *PAO1 have identified a gene called *arr *which is involved in tobramycin signaling at SIC [14] which results in an induction of biofilm formation. This gene encodes for an inner membrane protein and it was postulated to respond to tobramycin and affect surface appendages which are important for biofilm formation. The mode of action of Arr is currently unknown but it is believed to occur via the second messenger c-di-GMP since *arr *mutants are less active in degrading c-di-GMP [14]. It is currently unknown whether reduction of AHL levels by tobramycin signaling occurs via *arr*.

The tobramycin signaling reported here requires the interaction of tobramycin with the ribosome since ribosomal protection by methylation using the NpmA, RmtA and RmtC methyltransferases relieves signaling since SICs of tobramycin no longer affect the RhlI/R system. Tobramycin blocks the peptide exit channel by binding to the A1408 residue of 16 S rRNA and the three methyltransferases specifically methylate nucleotides A1408 [23] and G1405 [33,34] respectively, within the decoding A site in 16 S rRNA, no longer allowing tobramycin binding. Interestingly azithromycin signaling at SIC in strain PAO1 also requires interaction with the ribosome since similar experiments demonstrated that the ErmAB methyltranserase which blocks azithromycin interaction with the 23 S rRNA stops signalling at SICs [39]. These observations indicate that antibiotic signaling requires interaction with the ribosome possibly meaning the regulatory cascade is initiated at the ribosome.

## Conclusions

In summary this study has further highlighted the presence of antibiotic signaling in *P. aeruginosa *and that different strains can have a different response. The role of antibiotics as signaling molecules now needs to be further evidenced by unraveling the molecular mechanisms of the cascade identifying primary response components which trigger the regulatory cascade. Understanding the tobramycin response by *P. aeruginosa *will have important implications in the mode of utilization for human use of this very important antibiotic.

## Competing interests

The authors declare that they have no competing interests.

## Authors' contributions

FB performed all the experimental work and was also involved in manuscript writing. VV and GMV were involved in supervision of experimental design and data analysis, coordinated the study and drafted the manuscript. All authors read and approved the manuscript.

## Pre-publication history

The pre-publication history for this paper can be accessed here:

http://www.biomedcentral.com/1471-2334/10/148/prepub
